# Cell cycle responses to Topoisomerase II inhibition: Molecular mechanisms and clinical implications

**DOI:** 10.1083/jcb.202209125

**Published:** 2023-11-13

**Authors:** Tanya N. Soliman, Daniel Keifenheim, Peter J. Parker, Duncan J. Clarke

**Affiliations:** 1 Barts Cancer Institute, Queen Mary University London, London, UK; 2 Department of Genetics, Cell Biology and Development, University of Minnesota, Minneapolis, MN, USA; 3 The Francis Crick Institute, London, UK

## Abstract

DNA Topoisomerase IIA (Topo IIA) is an enzyme that alters the topological state of DNA and is essential for the separation of replicated sister chromatids and the integrity of cell division. Topo IIA dysfunction activates cell cycle checkpoints, resulting in arrest in either the G2-phase or metaphase of mitosis, ultimately triggering the abscission checkpoint if non-disjunction persists. These events, which directly or indirectly monitor the activity of Topo IIA, have become of major interest as many cancers have deficiencies in Topoisomerase checkpoints, leading to genome instability. Recent studies into how cells sense Topo IIA dysfunction and respond by regulating cell cycle progression demonstrate that the Topo IIA G2 checkpoint is distinct from the G2-DNA damage checkpoint. Likewise, in mitosis, the metaphase Topo IIA checkpoint is separate from the spindle assembly checkpoint. Here, we integrate mechanistic knowledge of Topo IIA checkpoints with the current understanding of how cells regulate progression through the cell cycle to accomplish faithful genome transmission and discuss the opportunities this offers for therapy.

## Teaser

Soliman et al. integrate recent molecular discoveries to bring insights and offer future directions to further our understanding of Topoisomerase IIA checkpoints.

## Introduction

Chromosome segregation errors result in aneuploidy that causes infertility, birth defects, and cancer (Klaasen and Kops, 2022). Aneuploidy has an enormous impact on human health: about 30% of miscarriages are due to aneuploidy; 1/1,000 live-born babies are aneuploid, suffering a variety of developmental disorders (see Brosens et al., 2022; McCoy, 2017); in adults, segregation errors drive cancer (Klaasen and Kops, 2022). A prominent cause of chromosome segregation errors is failure of cell cycle control mechanisms that ensure the fidelity of genome transmission, including controls that act upon entry into and progression through mitosis (Abbas et al., 2013; Potapova et al., 2013).

**Figure 1.**
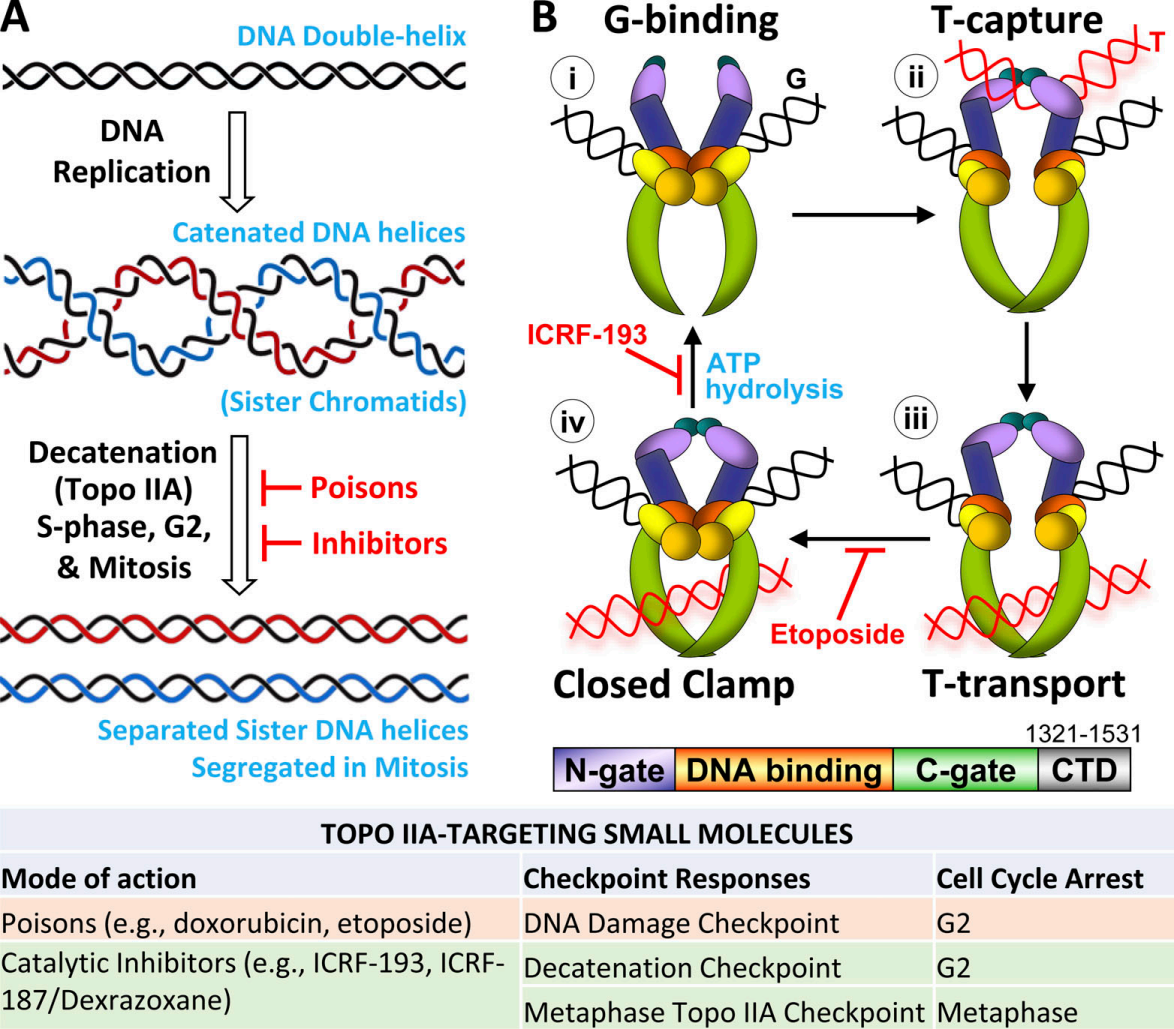
**Decatenation by Topo IIA is disrupted by small molecules. (A)** DNA replication produces catenated (entangled) sister chromatid DNA molecules. Topo IIA is highly conserved and is the only eukaryotic enzyme that can resolve catenations to permit the separation and segregation of sister chromatids during anaphase of mitosis. Decatenation is achieved progressively, being initiated during DNA replication, continuing in G2-phase, and being completed in mitosis. The activity of Topo IIA can be perturbed by small molecules categorized into two distinct classes dependent on their mode of action: poisons and catalytic inhibitors. **(B)** The unique SPR of DNA Topo II decatenates DNA helices. **(i–iv)** Top: SPR cycle: (i) G-segment double-helical DNA binds at the catalytic core (orange/yellow); (ii) T-segment DNA captured by the enzyme N-gate (purple), G-segment cleavage; (iii) T-segment transport; (iv) G-segment re-ligation—transient closed clamp enzyme conformation until ATP hydrolysis opens N-gate and C-gate (green). The SPR can be interrupted by small molecules at various points in the cycle including, but not limited to, those shown for Etoposide and ICRF-193 (red). The Topo II poison Etoposide blocks the re-ligation step, while the catalytic inhibitor ICRF-193 inhibits ATP hydrolysis. Bottom: Domain structure (colors match domains above). The CTD has not been crystalized. Table: Two categories of small molecules that target Topo IIA and their cell cycle consequences. One category, poisons, prevents G-segment ligation (step iii to iv in panel B), which generates double-stranded DNA breaks and triggers the G2-phase DNA damage checkpoint. Major chemical classes are anthracyclines (e.g., Doxorubicin, Epirubicin, and Daunorubicin) and Epipodophyllotoxins (e.g., Etoposide, Teniposide). The other category, catalytic inhibitors, traps Topo II on DNA, forming a closed clamp complex (iv in panel B) triggering the G2-phase decatenation and metaphase Topo IIA checkpoints. Bisdioxopiperazines are the most widely studied (e.g., ICRF-193, ICRF-187/Dexrazoxane, Sobuzoxane/MST-16). The focus of this review is cellular responses to catalytic inhibition, where cycle checkpoints are triggered by DNA–Topo IIA closed clamps. These structures arise naturally but are stabilized by treatment with catalytic inhibitors.

DNA replication produces physically entangled (catenated) sister DNA helices that must be resolved to allow chromosome segregation (Fig. 1 A; Cook, 1991). Topoisomerase IIA (Topo IIA) is the only enzyme that can decatenate these topological links between sister chromatids, rendering the enzyme and its unique strand passage reaction (SPR) essential for mitosis (Brown and Cozzarelli, 1979; Holm et al., 1985; Liu et al., 1979; Wang, 2002). When Topo IIA is perturbed, chromosomes can missegregate, leading to formation of micronuclei. Even a single micronucleus containing just one chromosome can cause genome instability via mechanisms such as chromothripsis, promoting cancer (Dahiya et al., 2022; De Marco Zompit and Stucki, 2021; Umbreit et al., 2020). It is critical for genome integrity that Topo IIA resolves all catenation before anaphase. Furthermore, because one class of Topo IIA inhibitors are workhorse cancer drugs (Nitiss, 2009), it has been important to understand the biochemical and structural basis of the SPR, which is targeted in cancer treatments (Dong and Berger, 2007; Ling et al., 2022; Vanden Broeck et al., 2021; Wendorff et al., 2012).

The SPR is performed by a symmetrical homodimeric Topo IIA holoenzyme requiring hydrolysis of two ATP molecules (Fig. 1 B; Abbas et al., 2013; Wang, 2002). Topo IIA makes a transient double-strand break (DSB) in one DNA helix, referred to as the gated-segment (G-segment), passes a second helix through the break (the transported-segment or T-segment), and then re-ligates the G-segment (Brown and Cozzarelli, 1979; Liu et al., 1979; Wang, 2002). The central region of the enzyme forms the catalytic core, which binds and cuts the G-segment allowing passage of the T-segment through the enzyme. While this intricate structural view of Topo IIA explains how DNA molecules are decatenated, to prevent erroneous chromosome segregation, control systems are needed to ensure decatenation is completed before anaphase (Andrews et al., 2006; Clarke et al., 2006; Downes et al., 1994; Furniss et al., 2013; Pandey et al., 2020).

## The Topo IIA G2-phase (decatenation) checkpoint

**Figure 2.**
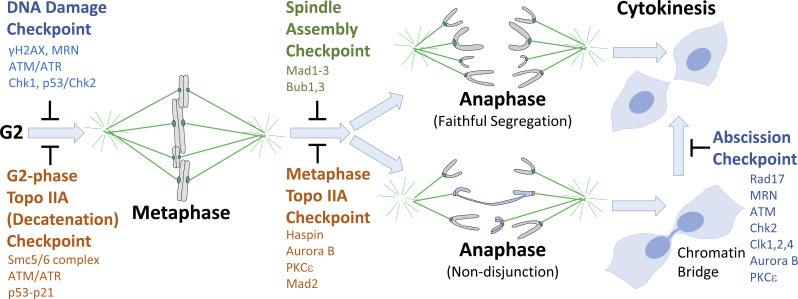
**Checkpoints regulating mitotic progression.** Topo IIA checkpoints operate in parallel with the G2 DDR and the SAC to regulate the G2-to-M and metaphase-to-anaphase cell cycle transitions. Key components required for each checkpoint response are shown. When non-disjunction occurs leading to an unresolved chromatin bridge, the abscission checkpoint delays cytokinesis until bridge resolution, after which PKCɛ phosphorylates Aurora B on S227 to permit abscission.

The DNA damage response (DDR) pathway is a G2 checkpoint responsible for arresting cells in G2 in response to DNA damage (Fig. 2). The DDR involves the activation of ataxia-telangiectasia mutated kinase (ATM)/ATM-related (ATR) and both Chk1 and p53/Chk2 pathways acting redundantly in G2 (see Lockwood et al., 2022) to trigger cell cycle arrest, allowing for DNA repair and subsequent cell cycle re-entry (Ciccia and Elledge, 2010). Topo IIA poisons, such as Etoposide, trap the covalent Topo IIA–DNA intermediate following strand passage, but prior to the DSB re-ligation step (see above, Fig. 1 B iii), triggering a DDR-related G2 arrest (Nam et al., 2010). This DNA damage–associated behavior has influenced perception of the Topo IIA–dependent checkpoint in G2 (Downes et al., 1994; Kaufmann and Kies, 1998), which likely reflects the physiological process of sensing ongoing post–S-phase decatenation of sister chromatids to avoid non-disjunction. Topo IIA responsive cell cycle controls seem to detect a stalled SPR intermediate known as the Topo IIA closed clamp (Fig. 1 B iv; Furniss et al., 2013; Pandey et al., 2020). The closed clamp is a natural structural conformation that exists transiently at the end of the normal enzyme cycle (Roca et al., 1994; Wang, 2002). Following re-ligation of the cut DNA helix (i.e., in the absence of any DNA damage), a Topo IIA closed clamp is trapped on DNA until both ATP molecules have been hydrolyzed, which allows opening of the N-terminal and C-terminal gates of the enzyme. This step must occur for Topo IIA to release from DNA and begin a new SPR cycle (Roca et al., 1994; Wang, 2002). The prolongation of this sensing checkpoint can be triggered pharmacologically by catalytic inhibitors of the ICRF-193 class, which block ATPase activity in this final step of the catalytic cycle of Topo IIA, preventing Topo IIA release from chromatin (Fig. 1 B iv). The distinction between Topo IIA poisons and catalytic inhibitors and the cell cycle responses they elicit is summarized in Box 1.

Box 1 Definitions and scope of the review

Topo II is the only enzyme in eukaryotes that can disentangle intertwined DNA duplexes, i.e., catenated DNA. Many inhibitors have been identified directed at Topo II (reviewed in Larsen et al., 2003), including those that suppress association to DNA, prevent ATP binding, block the re-ligation step, and inhibit the ATPase activity required for enzyme release from DNA (see scheme in Fig. 1). Pertinent to this review are the ligation inhibitors typically referred to as Topo II poisons that trap the enzyme–DNA intermediate, prevent re-ligation of the Topo IIA–implemented DSB, and hence trigger a DNA damage response (see text and Fig. 1 for further details). These agents, which include Etoposide, are used as cytotoxic agents in the treatment of some cancers. The focus of attention here is, however, on the ATPase inhibitors that trap Topo II on DNA at the end of its catalytic cycle; it is this class of inhibitors that trigger specific Topo II–dependent checkpoint delays in the cell cycle. These checkpoints act in G2, M, or at cytokinesis, reflecting the natural cellular monitoring of catenation by Topo II occupancy on chromatin, a process amplified by the inhibition of its catalytic activity. Our current understanding of the underlying controls operating in these checkpoints is reviewed here. The idiosyncrasies of these Topo II–dependent checkpoint controls may lend themselves to distinctive anticancer approaches as discussed.

In untransformed cells that display a sustained G2 arrest in response to ICRF-193, there is no sustained DNA damage as determined by γH2AX immunostaining; the limited extent of γH2AX staining on exit from S-phase is resolved shortly after S-phase in a Topo IIA–independent manner, and this resolution does not influence the ICRF-193–induced G2 arrest (Lockwood et al., 2022; it is noted that transformed cells that progress through the cell cycle despite ICRF-193 exposure typically undergo aberrant cell divisions triggering DNA damage). The ICRF-193–induced G2 arrest is dependent upon the expression of Topo IIA (Bower et al., 2010a; Furniss et al., 2013); knock-down of Topo IIA bypasses the arrest while leaving chromosome catenations (Bower et al., 2010a; Brownlow et al., 2014). A similar pattern of behavior occurs in budding yeast (Baxter and Diffley, 2008; Furniss et al., 2013). This provides evidence that the arrest is not simply triggered by a physical constraint on intertwined chromatin nor by some independent sensing of catenated chromatin, but is in fact relayed to the cell cycle machinery in a manner dependent upon Topo IIA and by inference its specific conformation and juxta-positioning on chromatin at G2 (i.e., post-replication). Topo IIA is in effect used both as the sensor of retained catenation and as the executor of its resolution. The existence of a Topo IIA–linked signaling pathway responsible for implementing this arrest (see below) formalizes this as a Topo IIA–dependent G2 checkpoint. As noted above, the purpose of this checkpoint is to ensure chromatin catenation-associated disjunction prior to M-phase entry (replication stress and associated non-disjunction do not trigger the same signaling relay) (recently reviewed in Saxena and Zou, 2022).

In early studies, specific regulators were shown to be involved in the implementation of the Topo IIA–dependent checkpoint, and these included p53 (Doherty et al., 2003; Kaufmann et al., 2002). Recent genome-wide screening has elucidated a more detailed signal relay responsible for implementing the Topo IIA–dependent G2 checkpoint. This has elaborated a pathway involving the DNA-interacting SMC5/6 complex subunits, the protein kinases ATM/ATR (acting redundantly), alongside the tumor suppressor p53 and the cdk inhibitor p21 (Deiss et al., 2019). Contrasting with the DDR (Stracker et al., 2009), this relay does not trigger activation of, or require, Chk1/2 (Deiss et al., 2019; Deming et al., 2001), although the downstream p53-p21 module in this checkpoint pathway overlaps with one of the redundant DDR pathways acting in G2 (see Lockwood et al., 2022). The upstream triggers define the distinctiveness of these G2 checkpoints with, for example, the SMC5/6 complex and Topo IIA proteins being essential for the ICRF-193–induced arrest while not impacting the DDR; the converse being true for DNA-dependent protein kinase (DNA-PK)–dependent sensing of chemically induced DSBs (reviewed in Jackson and Bartek, 2009). The requirement for the SMC5/6 complex in the Topo IIA–dependent checkpoint has been demonstrated by manipulation of subunit expression (all six mammalian subunits; SMC5–6 and NSMCE1–4) in cells and also verified in cells derived from a patient with a germline mutation in the NSMCE2 subunit of the complex (Deiss et al., 2019). NSMCE2 is an E3 ligase responsible for the ICRF-193–induced SUMOylation of a non-canonical site, K1520, at the C-terminus of Topo IIA, and defects in this activity lead to a failure to sustain a G2 arrest in response to ICRF-193 (Deiss et al., 2019).

The implementation of the Topo IIA checkpoint in G2 is associated with the accumulation of Topo IIA and the SMC5/6 complex in promyelocytic leukemia protein (PML) bodies. The colocalization of these proteins is likely important in promoting NSMCE2-directed SUMOylation of Topo IIA, although no formal evidence of higher-order complex formation/physical contact has yet been seen within these nuclear structures. Nevertheless, given the pattern of S-phase chromatin loading and M-phase unloading of the SMC5/6 complex (Gallego-Paez et al., 2014; Tsuyama et al., 2006), a role in supporting the sensing and completion of post-replicative sister chromatid disjunction is an attractive proposition. While there are numerous roles assigned to the SMC5/6 complex (Palecek, 2018), it is of interest that based on separation of function alleles, the essential role for the related SMC5/6 complex in yeast is manifest specifically in G2/M (Menolfi et al., 2015).

Interestingly, in transformed cells engaging alternative lengthening of telomeres (ALT cells), ICRF-193 induces accumulation of Topo IIA and the SMC5/6 complex in a subset of PML bodies, termed APBs (ALT-associated promyelocytic leukemia nuclear bodies), and this is associated with a G2 arrest (Potts and Yu, 2007). However, in this context, the arrest is driven in a Chk1-dependent manner and is independent of p53 (Lockwood et al., 2022), consistent with the association of p53 inaction in ALT tumors (Barthel et al., 2017). The arrest requires the RecQ-like helicase Bloom's syndrome protein (BLM) (Lockwood et al., 2022), and perhaps, related to this, it has been shown that Topo IIA can be found associated with BLM and telomere-binding protein TRF2 in ALT cells (Bhattacharyya et al., 2009; Lockwood et al., 2022). It is likely that Topo IIA contributes to the resolution of the recombination intermediates associated with this specific telomere elongation process (see, for example, Hou et al., 2022), and hence that this is sensed when it becomes trapped on the telomeric structures by ICRF-193. The implication is that the signaling relays triggering arrest downstream of SMC5/6-Topo IIA are exquisitely a function of the context, i.e., the corecruited regulators. This context-dependent behavior provides a rationalization for the identification of other players that have been identified in ICRF-193–induced G2 arrest(s): BRCA1, WRN, MDC1, Plk1, MCPH1, and Chk1 (Arroyo et al., 2019, 2020; Deming et al., 2001, 2002; Lou et al., 2005; Luo et al., 2009; Robinson et al., 2007; Spoerri et al., 2016).

While untransformed cells can engage a long-term G2 arrest in response to ICRF-193, cells have also been reported to trigger a short-term, caffeine-sensitive (requiring ATM or ATR) delay in G2 progression even in the absence of p53 (Kaufmann et al., 2002). It has been established that this transient delay is distinguished from the DDR and requires Topo IIA (Kaufmann et al., 2002). A distinctive element relaying this response is the phosphorylation of Topo IIA on S1524 and the recruitment of MDC1 through its BRCA1 C-terminal (BRCT) domain (Luo et al., 2009). MDC1 is itself required for the DNA damage response; however, the Topo IIA–MDC1 complex is not (Luo et al., 2009). Whether these responses involve the SMC5/6 complex remains to be determined, although it would be attractive to posit the bifurcation of signals from SMC5/6-Topo IIA leading to a G2 delay on the one hand (p53 independent) and a program of long-term arrest and senescence on the other (p53 dependent). The differential localization of these proteins (e.g., in or out of PML bodies) and the potential distinctive coassociation of other regulators perhaps determine these outputs much as ALT cells display distinctive ICRF-193–induced downstream events (discussed above).

Irrespective of the details that have yet to be resolved, the evidence indicates that there is a checkpoint in G2 that monitors the Topo IIA closed clamp occupation of chromatin. The specific circumstances evidently impact the particular pathways engaged but the implication remains the same; if Topo IIA in this conformation is sensed on chromatin associated with the SMC5/6 complex, then there is a delay to G2 progression. The existence of these regulatory processes suggests that in the absence of any experimental manipulation, the steady-state association of Topo IIA with chromatin is exploited by cells to sense ongoing decatenation, the associated potential for non-disjunction, and hence the need for cell cycle delay and catenane resolution.

## Engagement of the metaphase Topo IIA checkpoint is distinct from the spindle assembly checkpoint (SAC) and is associated with promotion of catenation resolution

The mitotic apparatus, the spindle and kinetochores, is mechanically complex, but without cell cycle controls, chromosome segregation is error prone. In early mitosis, the SAC senses chromosome biorientation, deciding when anaphase can proceed accurately (Foley and Kapoor, 2013; Lara-Gonzalez et al., 2021; Musacchio, 2015). These controls sense microtubule attachments to kinetochores and tension forces to ascertain if biorientation has been achieved (reviewed in Chacón et al., 2014). In parallel, cells monitor Topo IIA to ensure that the sister chromatids can be efficiently separated before chromosome segregation is initiated in anaphase (Fig. 2). Like the G2-phase Topo IIA checkpoint, this metaphase Topo IIA checkpoint seems to be activated by Topo IIA–DNA complexes, recruiting cell cycle regulators to chromosomes to stop cells initiating anaphase. These Topo IIA complexes can be generated experimentally, either by blocking ATP hydrolysis with catalytic Topo IIA inhibitors (e.g., ICRF-193) or genetically by mutating residues of Topo IIA leading to perturbed ATPase hydrolysis (Brownlow et al., 2014; Clarke et al., 2006; Iwai et al., 1997; Kelly et al., 2020; Skoufias et al., 2004; Toyoda and Yanagida, 2006).

**Figure 3.**
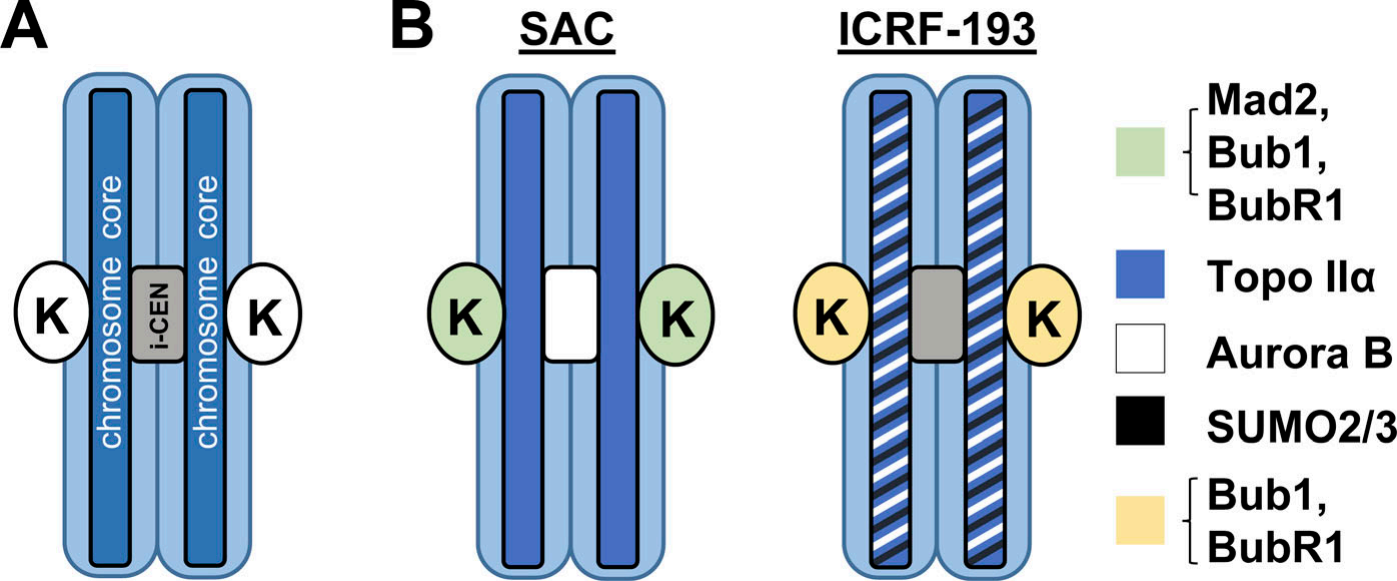
**Chromosomal localization of mitotic checkpoint regulators. (A)** Cartoon showing relative location in mitosis of sister kinetochores (K; ovals), sister chromatid cores (chromosome cores), and the inner centromere (i-CEN; gray). The entire chromosome arm width is indicated by light blue–shaded regions. **(B)** Localization patterns of Mad2, Bub1, BubR1, Aurora B, Topo IIA, and SUMO2/3 in mitosis after nocodazole treatment (SAC activation) versus ICRF-193 treatment (Topo IIA checkpoint activation). Mad2 localizes to kinetochores and Aurora B remains at the inner centromere after nocodazole treatment, while SUMO2/3 is distributed diffusely. After a brief (30 min) ICRF-193 treatment in metaphase, Aurora B is recruited away from centromeres to the chromosome cores along with SUMO2/3. Consistent with chromosome biorientation being maintained, Mad2 is not recruited to kinetochores, but Bub1 and BubR1 are retained, dependent on PKCɛ.

The mechanism of the metaphase Topo IIA checkpoint is at least partially conserved from yeast to human cells (Andrews et al., 2006; Clarke et al., 2006; Furniss et al., 2013; Pandey et al., 2020), revealing that it is a fundamental aspect of mitotic regulation. Studies in human cells initially used catalytic inhibitors such as ICRF-193 to inhibit Topo IIA ATPase activity in metaphase, which resulted in delayed anaphase onset (Brownlow et al., 2014; Clarke et al., 2006; Iwai et al., 1997; Kelly et al., 2020; Pandey et al., 2020; Skoufias et al., 2004; Toyoda and Yanagida, 2006). In yeast genetic screens, *top2* mutants that activate the metaphase Topo IIA checkpoint were isolated and found to be defective in ATPase activity (Andrews et al., 2006; Furniss et al., 2013). Thus, the yeast *top2* mutants genetically mimic ICRF-193 treatment in human cells. Subsequent studies confirmed that the metaphase Topo IIA checkpoint mechanism is distinct from the canonical SAC (Andrews et al., 2006; Brownlow et al., 2014; Clarke et al., 2006; Furniss et al., 2013; Kelly et al., 2020; Pandey et al., 2020; Skoufias et al., 2004). Core components required for metaphase Topo IIA checkpoint activation are Haspin kinase, Aurora B, and protein kinase Cε (PKCɛ; Brownlow et al., 2014; Pandey et al., 2020). Both SAC and metaphase Topo IIA checkpoints ultimately activate Mad2 to prevent anaphase onset but by using distinct mechanisms (Andrews et al., 2006; Skoufias et al., 2004; Toyoda and Yanagida, 2006). The SAC recruits Mad2 to kinetochores where it is activated and then released. The SAC therefore requires intact kinetochores for activation (see Rieder et al., 1995; Tavormina and Burke, 1998). In contrast, Mad2 does not localize to kinetochores when the metaphase Topo IIA checkpoint is active (Brownlow et al., 2014; Skoufias et al., 2004; Toyoda and Yanagida, 2006; Fig. 3), and (at least in yeast) kinetochores are not required for metaphase Topo IIA checkpoint activation (Andrews et al., 2006; Skoufias et al., 2004; Toyoda and Yanagida, 2006). In summary, while the SAC monitors chromosome spindle attachment, the metaphase Topo IIA checkpoint monitors ongoing Topo IIA (closed clamp) occupation of chromatin to ensure sister chromatids can separate in anaphase (Furniss et al., 2013).

Factors required for the metaphase Topo IIA checkpoint in human cells were initially identified based on analysis of the conserved mechanism in yeast (Andrews et al., 2006; Furniss et al., 2013), which among other factors, identified Ipl1 (yeast ortholog of Aurora B kinase) as an essential component. Anaphase onset is delayed when the Topo IIA catalytic inhibitor ICRF-193 is added to metaphase human cells, but this was bypassed by Aurora B inhibitors (Pandey et al., 2020). At the same concentrations, these inhibitors could not bypass the SAC (induced with nocodazole that depolymerizes microtubules), demonstrating the SAC and metaphase Topo IIA checkpoint have distinct mechanisms (Pandey et al., 2020). In these experiments, because ICRF-193 was added to metaphase cells, chromosome biorientation had already occurred, satisfying the SAC (Pandey et al., 2020). This might mean there is a window of time in metaphase after SAC inactivation when the Topo IIA checkpoint can still be engaged. Nevertheless, a fraction of cells proceeds quickly into anaphase when ICRF-193 is added in metaphase, suggesting those cells had already passed a commitment point to allow the metaphase–anaphase transition (Pandey et al., 2020).

Given that ICRF-193 does not activate the canonical SAC, an important question is how Aurora B is activated to induce the metaphase Topo IIA checkpoint. Clues came from the fact that ICRF-193 induces SUMOylation of the Topo IIA C-terminal domain (CTD) specifically on mitotic chromosomes and that in both interphase and mitotic cells, SUMOylation initiates repair pathways that have evolved to remove trapped Topo IIA complexes from DNA (Agostinho et al., 2008; Hassebroek et al., 2020; Isik et al., 2003; Schellenberg et al., 2017; Tian et al., 2021; Xiao et al., 2003; Yoshida et al., 2016). This led to the prediction that SUMOylation of Topo IIA would also activate the metaphase Topo IIA checkpoint to provide extra time for removal of the trapped complexes and for decatenation before anaphase onset. Consistent with this hypothesis, engineered human cells where endogenous Topo IIA is replaced with a mutant lacking three conserved SUMOylated lysines (Topo II-3KR) were partially defective in metaphase Topo IIA checkpoint activation (Pandey et al., 2020), demonstrating that SUMOylation in response to ICRF-193 is likely involved in Aurora B activation. The partial metaphase Topo IIA checkpoint bypass may indicate that additional lysines in the CTD are SUMOylated in the presence of ICRF-193, consistent with other studies (see Table 1; Antoniou-Kourounioti et al., 2019; Ryu et al., 2010; Schellenberg et al., 2017; Tian et al., 2021).

**Table 1.** Topo IIA SUMOylation site mutants and phenotypes

Species	Lysine(s)	Phenotypes/Functions	References
Yeast (*S. cerevisiae*)	1220 1246 1247 1277 1278	SUMOylation of these lysine residues is implicated in: (1) regulating sister centromere cohesion, (2) recruitment of Ipl1 (yeast ortholog of Aurora B) to centromeres in mitosis, and (3) inducing ubiquitination of Top2-DNA cleavage complexes.	(1) Bachant et al. (2002) (2) Edgerton et al. (2016) (3) Sun et al. (2020)
	1220 1246 1277	Required for stable maintenance of minichromosomes. Triple K-to-R mutant stabilizes dicentric chromosomes, indicating reduced kinetochore function.	Takahashi et al. (2006)
Frog (*X. Laevis* egg extracts)	660	Inhibits Topo IIA decatenation activity. Regulates resolution of centromeric catenations in mitosis.	Ryu et al. (2010)
	1235 1276 1298	Recruitment of (1) Claspin, (2) Haspin, and Aurora B to mitotic chromosomes.	(1) Ryu et al. (2015) (2) Yoshida et al. (2016)
Human	662	Topo IIA-K662R has reduced Topo IIA centromere localization and increased chromosome segregation defects.	Antoniou-Kourounioti et al. (2019)
	1228 1240	SUMOylation by ZATT/ZNF541 ligase induced by replication fork stalling (hydroxyurea treatment in S-phase) promotes fork reversal and recruitment of PICH DNA translocase.	Tian et al. (2021)
	1240	Topo IIA-K1240R mutant has reduced Topo IIA centromere localization.	Antoniou-Kourounioti et al. (2019)
	1240 1267 1286	Triple (K-to-R) mutant reduces Aurora B recruitment to mitotic chromosomes induced by ICRF-193 and partially bypasses the metaphase Topo IIA checkpoint.	Pandey et al. (2020)
	1520	SUMOylated by NSE2 (Smc5/6 complex) E3 ligase induced by ICRF-193. Topo IIA-K1520R mutant increases chromosome segregation defects but does not affect the G2-phase Topo IIA checkpoint, unlike depletion of Smc5/6 complex components, which does lead to a failure to sustain G2 arrest in response to ICRF-193.	Deiss et al. (2019)

**Figure 4.**
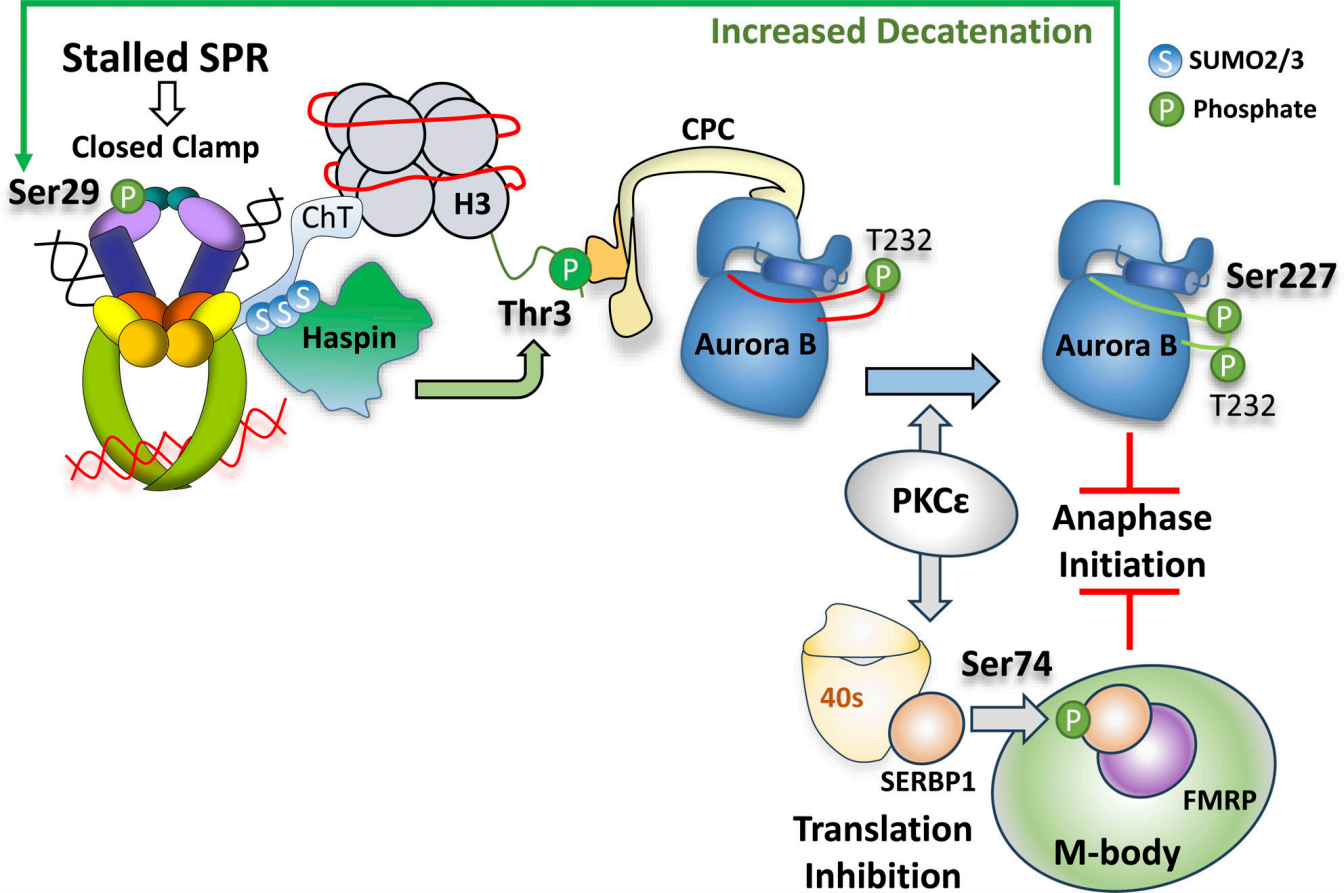
**Metaphase Topo II checkpoint model.** Closed clamp Topo II complexes are SUMOylated, recruiting Haspin via its SIMs to Topo IIA–bound nucleosomes. Haspin generates H3T3p, which recruits CPC–Aurora B. PKCɛ phosphorylates Aurora B–S227, inducing a substrate-specificity switch required for metaphase checkpoint activation and increased Topo IIA activity. In parallel, PKCɛ phosphorylates SERBP1, which is sequestered in M-bodies, repressing translation, needed for successful cell division.

The evidence implicating Topo IIA SUMOylation in the metaphase checkpoint activation is important because it provides a direct link to Aurora B activation. In yeast and Xenopus egg extracts, Topo IIA SUMOylation recruits Haspin kinase to chromosomes in mitosis (Edgerton et al., 2016; Yoshida et al., 2016), and Haspin generates a binding site for Aurora B by phosphorylating histone H3 at Threonine 3 (H3T3-phos; Fig. 4). Haspin has SUMO interacting motifs (SIMs) that mediate a direct interaction with SUMOylated Topo IIA, explaining how trapped Topo IIA complexes recruit Haspin to chromosomes (Yoshida et al., 2016). H3T3 is the only known substrate of Haspin, and when phosphorylated, it interacts directly with Survivin, a subunit of the chromosome passenger complex (CPC) of which Aurora B is also a component (Dai et al., 2005; De Antoni et al., 2012; Kelly et al., 2010; Wang et al., 2010, 2011, 2012). Crucially, Haspin inhibitors were found to bypass metaphase Topo IIA checkpoint activation, like Aurora B inhibitors (O’Connor et al., 2015; Pandey et al., 2020). Consistent with this cascade being responsible for metaphase Topo IIA checkpoint activation, both Haspin kinase activity and Topo IIA SUMOylation are required for efficient recruitment of Aurora B to chromosomes in mitotic cells treated with ICRF-193. Strikingly, Aurora B was recruited to chromosome cores, where trapped Topo IIA complexes are most abundant (Fig. 3). This contrasts with the localization of Aurora B at inner centromeres and kinetochores when the SAC is active (Broad et al., 2020; Campbell and Desai, 2013; Carmena et al., 2012; Dai et al., 2006; Petsalaki and Zachos, 2016; Yamagishi et al., 2010; Fig. 3). Finally, ICRF-193 treatment in mitosis also induced a dramatic increase of SUMO2/3 at chromosome cores. Chromosome cores are the central region of chromatid arms where Topo IIA is concentrated, and indeed SUMO2/3, Aurora B, and Topo IIA localized with the same pattern at the cores after ICRF-193 treatment (Fig. 3; Pandey et al., 2020). Altogether, the evidence supports a model where the metaphase Topo IIA checkpoint is activated by SUMOylated Topo IIA, which recruits Haspin to then recruit Aurora B (Fig. 4).

Additional evidence has indicated that nucleosome binding of Topo IIA may facilitate activation of the metaphase Topo IIA checkpoint. Aurora B recruitment seems to rely on H3T3 phosphorylation by Haspin, leading to the prediction that Haspin must not only be recruited to chromosomes but must be in close proximity to nucleosomes. The discovery of a nucleosome binding region of Topo IIA, the chromatin tether (ChT) domain (Lane et al., 2013), could provide this missing link. Deletion of the ChT results in chromosome segregation errors, the expected phenotype if the checkpoint requires the ChT to efficiently enact Haspin-mediated H3T3 phosphorylation (Sundararajan et al., 2023). It will be important to determine if the ChT is required for the activation of the checkpoint.

Another major gap in understanding is how Aurora B activates the metaphase Topo IIA checkpoint via Mad2 activation. Because there is no direct connection between Aurora B and Mad2, the relevant substrates of Aurora B during metaphase Topo IIA checkpoint activation are unknown. There may be novel Aurora B substrates involved since Aurora B is recruited to a distinctive location at chromosome cores where established kinetochore substrates are not known to be present. Further, metaphase Topo IIA checkpoint activation requires PKCε, which triggers a switch in Aurora B substrate selection (Brownlow et al., 2014; Kelly et al., 2020). PKCε phosphorylates a second site in the activation loop of Aurora B (S227), and this specifically occurs in the chromosome-associated fraction of Aurora B in mitosis upon metaphase Topo IIA checkpoint activation (Fig. 4; Kelly et al., 2020). S227 phosphorylation induces a significant conformational change in the activation loop, and as a result, there is a dramatic change in Aurora B substrate specificity (Kelly et al., 2020; Pike et al., 2016). Importantly, knock-down of the WT protein and replacement with an Aurora B S227A mutant led to increased chromosome segregation errors and ultrafine DNA bridges (UFBs) in anaphase and was also deficient in metaphase Topo IIA checkpoint activation. This evidence demonstrates that the Aurora B substrate specificity switch is essential for proper engagement of the metaphase Topo IIA checkpoint, but the relevant Aurora B substrate(s) are not known. The Aurora B specificity switch has an additional crucial consequence: increased decatenation. The specificity switch promotes Aurora B phosphorylation of Topo IIA at Serine 29, which substantially increases Topo IIA activity facilitating resolution of this topological block to cell division (Fig. 4; Kelly et al., 2020). It appears that the sensing, arrest, and resolution are integrated into a coherent set of events that are by their very nature self-limiting.

Given that the effectors of Aurora B remain to be identified, further studies are needed to determine how Mad2 is activated. The identity of the Topo IIA SUMO E3 ligase that activates the checkpoint is also unknown. Several E3 ligases have been shown to SUMOylate Topo IIA, including PIASy and RanBP2 (Azuma et al., 2005; Dawlaty et al., 2008; Díaz-Martínez et al., 2006; Navarro and Bachant, 2008), ZNF451/ZATT (Schellenberg et al., 2017) and, as described above, NSMCE2 (Deiss et al., 2019). ZATT is known to SUMOylate Topo IIA in interphase and functions in removal of trapped Topo IIA complexes from DNA (Riccio et al., 2020; Schellenberg et al., 2017; Tian et al., 2021). NSMCE2 is a component of SMC5/6 complexes that can SUMOylate Topo IIA, and as discussed above is known to be required for the G2-phase Topo IIA checkpoint activated by ICRF-193 (Deiss et al., 2019). It will be important to determine which of these E3 ligases act in mitosis and which of the known Topo IIA SUMOylation sites can recruit Haspin to chromosomes. It is already evident that SUMOylation of different lysine residues has different outcomes (see Table 1).

## Bypassing Topo IIA checkpoints

Repair mechanisms have evolved to disperse trapped Topo IIA complexes (Agostinho et al., 2008; Hassebroek et al., 2020; Isik et al., 2003; Schellenberg et al., 2017; Tian et al., 2021; Xiao et al., 2003; Yoshida et al., 2016); however, the extent to which these operate physiologically to bypass or exit the Topo IIA checkpoints and hence impact the timing of these cell cycle delays remains to be determined. By contrast, there is clear evidence for somatic changes influencing checkpoint function and consequently triggering downstream events. Cells with a defective Topo IIA–dependent G2 checkpoint can enter mitosis with an excessive amount of unresolved DNA catenanes, and if unresolved prior to anaphase entry, non-disjunction is manifest in Plk1-interacting checkpint helicase (PICH)-positive UFBs during anaphase (Wang et al., 2008). Unresolved, this non-disjunction leads to chromosome segregation errors, division failure, and aneuploidy. It is noted that the retention of some catenated DNA on entry into mitosis, particularly at the centromere, appears to be required to maintain chromosome structure (Bauer et al., 2012; Bower et al., 2010a). Hence, a low degree of catenation in mitosis may be “normal.” The monitoring and resolution of this residual centromeric catenation may be linked to the timing of anaphase entry/progression, presumably under the control of Haspin–Aurora B responsible for the Topo IIA metaphase checkpoint (see above).

There is evidence that the resolution of excessive catenation appears to engage additional/distinct mitotic pathways that are determined by the specific G2 trigger. This is exemplified by the engagement of PKCε during mitosis, where loss of the p53-dependent checkpoint pathway in G2 is associated with a subsequent dependence on PKCε for effective cell division (Brownlow et al., 2014; Kelly et al., 2020; Lockwood et al., 2022; Martini et al., 2018). By contrast, in ALT cells, where p53 is dysfunctional and Chk1 is the key downstream relay for the G2 arrest, bypassing this ICRF-193–induced engagement of the G2 checkpoint does not engage PKCε and by inference requires distinct regulators to facilitate subsequent mitotic delay and resolution (Lockwood et al., 2022). The extent to which Haspin and Aurora B are common to all mitotic Topo IIA–associated delays, while PKCε and other regulators are idiosyncrasies of the context, remains to be determined. Likewise, it remains to be determined whether Topo IIA activation (Kelly et al., 2020), translational repression (Martini et al., 2021), and Aurora B S227 phosphorylation (Pike et al., 2016) are required in all circumstances of mitotic delay, and if so what kinases other than PKCε can execute these actions (see Fig. 4).

Bypassing the Topo IIA checkpoints engenders DNA bridging, which persists into telophase and cytokinesis, activating the Aurora B–dependent abscission checkpoint to delay cytokinesis (see Fig. 2; Bhowmick et al., 2019; Steigemann et al., 2009). Interestingly, it has been shown very recently that the sensing of DNA bridging at the abscission checkpoint can be effected through the detection of trapped Topo IIA–DNA cleavage complexes in the DNA knots that form juxtaposed to the midbody (Petsalaki et al., 2023). This trapping is distinct from the closed clamp conformer associated with G2- and M-phase Topo IIA checkpoints, and the proximal signals affecting downstream events remain to be elucidated. Nevertheless, it is established that upon sensing DNA trapped in the cytokinetic furrow, Aurora B signaling is sustained, maintaining a stabilized intercellular canal (Steigemann et al., 2009) and preventing localization of the ESCRTIII complex, in particular CHMP4C, to the midbody ring and site of abscission (Carlton et al., 2012; Pike et al., 2016; the ESCRT machinery is involved in multiple aspects of membrane remodeling; see Olmos, 2022). This is reported to be antagonized by the UFB binding protein, RIF1, recruiting PP1 to unresolved DNA bridges during cytokinesis (Bhowmick et al., 2019). This counters the action of Aurora B to delay abscission and allow for bridge resolution. Exit from this arrest is associated with phosphorylation of Aurora B on S227 by a PKCε-14.3.3 complex at the midbody (Watson et al., 2021), switching specificity toward S165 on the CPC subunit, Borealin (Pike et al., 2016). The extent to which PP1 and the S227 kinase antagonism acts alongside CHMP4C (Carlton et al., 2012) and clk1/2/4 (Petsalaki and Zachos, 2016) to determine timing is unclear. It will be of interest to establish whether PP1 inactivation/mislocalization triggers Aurora B S227 and/or Borealin S165 phosphorylation, and where PKCε is not required (e.g., in ALT cells, see above), what acts to trigger S227 phosphorylation or is there an alternative route for abscission checkpoint exit? Ultimately, if bridging cannot be resolved, cells will fail cytokinesis through collapse of the midbody resulting in a binucleated cell or cells will divide despite the bridge leading to DNA breakage, accumulation of DNA damage, and ultimately aneuploidy (see, for example, Thoresen et al., 2014).

Defective decatenation checkpoints have been observed in a number of tumor types including lung (Lockwood et al., 2022; Nakagawa et al., 2004), melanoma (Brooks et al., 2014a, 2014b), colon (Jain et al., 2015), bladder (Doherty et al., 2003), and breast (Bower et al., 2017). Loss of the Topo IIA (decatenation) checkpoint in G2 correlates with inactivation or loss-of-function of p53 (Doherty et al., 2003; Kaufmann et al., 2002; Lockwood et al., 2022), known to be a common feature of not only these tumor types but more broadly; *TP53* is mutated in >50% of all human tumors (Bykov et al., 2018). A variety of effectors had been implicated in the G2-phase Topo IIA checkpoint response prior to wider genome screening, including BRCA1 (Lou et al., 2005), Chk1 (Robinson et al., 2007), WRN (Franchitto et al., 2003), and ATM (Bower et al., 2010b). ATM appears to act redundantly with ATR in normal epithelial cells (Deiss et al., 2019); however, it remains likely that particular transformed cell models exploit distinct arrest pathways as discussed above. Indeed, the identification of Chk1 as a requirement for ICRF-193–induced G2 arrest in DT40 cells (Robinson et al., 2007) would suggest operation of the ALT pathway in these cells; despite expressing telomerase there is evidence that this is the case (O’Hare and Delany, 2011). The implication is that for catalytic inhibition of Topo IIA, there may be a number of different conditional dependencies in transformed cells that are particular to the transformation. This poses a challenge in exploiting these pathways but is equally an opportunity for highly selective interventions.

## Opportunities for intervention

In implementing stress-associated cell cycle checkpoints, the uniqueness of the upstream triggers and the potential for a high degree of selectivity offers some interesting opportunities clinically (Box 2). Since “normal” cells typically do not expose themselves to the lower fidelity, Topo IIA–dependent controls operating in M-phase and at abscission, then in some cancers where the G2 checkpoint is dysfunctional (e.g., *TP53* mutant, discussed above), there are druggable targets that are unusually engaged in these conditional pathways. These could be exploited in a synthetic lethal approach alongside catalytic Topo IIA inhibitors. There is already good evidence that targeting tumors with a defective Topo IIA–dependent G2 response may be a viable therapeutic strategy. Checkpoint deficient cell lines including hepatocellular and pancreatic ductal adenocarcinoma (PDAC) (Lee et al., 2022) and colorectal cancer (Jain et al., 2015) lines have been reported to be sensitive to Topo IIA catalytic inhibition alone, with cells undergoing a catastrophic mitosis after being unable to decatenate their DNA. Why these cell types display this sensitivity is not yet understood, although a complete analysis of Topo IIA–associated checkpoint effector expression has yet to be reported. Melanoma cell lines that have a defective G2 decatenation checkpoint have been reported to be dependent on PI3K signaling and are sensitive to the PI3Kα,γ, and mTOR inhibitor PF-05212384 (Brooks et al., 2014b). Again, how, if at all, this dependency relates to Topo IIA checkpoint effector expression is unknown.

Box 2 Context of checkpoint interventions in cancer

There are many cytotoxic agents, including the Topo IIA poisons, deployed in cancer treatments. These drugs induce DNA damage, which in cycling cells causes the malfunction of DNA replication, associated disjunction errors, failed cell division, and cell death. The narrow therapeutic window for these agents is a consequence of the rapid division of cancers relative to most tissues. Nevertheless, rapidly turning over tissues, e.g., gut and bone marrow, are associated with the “side effects” of such treatments. More selective approaches to the blockade of cell division through the specific targeting of cell cycle controls and checkpoints have promised greater selectivity and a better therapeutic index compared with these general DNA-damaging agents. This is the case for the cdk4/6 inhibitor Palbociclib, which is FDA-approved for deployment as a combination therapy in HR+ advanced breast cancer (Du et al., 2020). While there has been evidence of poor tolerance for some other interventions of this type, the targeting of the cell cycle regulator Aurora B, for example, still offers promise (see Borah and Reddy, 2021). Related approaches that draw upon dysfunctional properties of tumor cells may also offer opportunities for intervention, and given the frequent loss of the G2 Topo IIA checkpoint in tumor cells, the downstream reliance on M-phase and anaphase checkpoints to resolve residual catenation yields the potential for a good therapeutic index in targeting these cell cycle controls.

Topo IIA catalytic inhibitors are currently used in clinical practice alongside anthracycline chemotherapeutics (Marty et al., 2006). In this setting, they provide a cardioprotective function as ROS scavengers (Lebrecht et al., 2007) or by targeting Topo IIB (Deng et al., 2014; Jirkovský et al., 2021). Use of Topo II catalytic inhibitors, for example, dexrazoxane (ICRF-187), in targeting the Topo IIA–dependent G2 checkpoint has not been assessed clinically (either as a single agent or in combination), but it is known to have a tolerable toxicity profile in patients (Macedo et al., 2019). Combining dexrazoxane with targeting the metaphase Topo IIA checkpoint may present a new therapeutic opportunity for *TP53* mutant cancers. Topo IIA inhibition would arrest normal, healthy cells in the surrounding tissues in G2-phase, while the checkpoint-deficient tumor cells would be primed for targeting the checkpoint relay engaged in metaphase. Known targets of the metaphase pathway include PKCε (Brownlow et al., 2014), Aurora B (Pandey et al., 2020), and PICH (Nielsen et al., 2015); BLM helicase (Nguyen et al., 2013; Russell et al., 2011) and Haspin (Amoussou et al., 2018; Huertas et al., 2012) could also be actionable targets in Topo IIA–dependent G2 checkpoint deficient tumors.

To date, no combinations drawing upon these findings have been tested in patients, and although it is the case that previously available PKCε inhibitors lack the desired specificity for clinical use (Roffey et al., 2009), new-generation inhibitors may overcome this limitation (Blasio et al., 2018). PKCε appears to be engaged in multiple pathways; however, at least in unstressed laboratory mice, its complete absence is tolerated (Castrillo et al., 2001), suggesting that its inhibition would not engender on-target toxicity. Beyond p53 loss-of-function, which tumor types might offer themselves to a combined PKCε/Topo IIA inhibitor attack remains to be determined. First-generation Aurora B inhibitors have proven to be too toxic (Kovacs et al., 2023); however, more recent inhibitors show promise (Borah and Reddy, 2021). Again, beyond p53 status, it is not clear where deployment of a combination treatment might be most effective. Targeting PICH may overcome the potential issues of undesired on-target/off-pathway consequences. PICH is engaged in resolution of non-disjunction structures in anaphase as is the BLM helicase (Rouzeau et al., 2012). PICH acts in part to disperse SUMOylated Topo IIA foci on chromatin (Hassebroek et al., 2020). Notably, PICH-deficient cell lines are sensitive to Topo IIA inhibition and become binucleated (Nielsen et al., 2015), while PICH depletion in triple-negative breast cancer cells leads to non-disjunction, cytokinesis failure, and ultimately apoptosis after mitotic catastrophe (Huang et al., 2019). It has also been reported that aneuploid or chromosomally unstable tumors, as might be driven by aberrations/inhibition of Topo IIA–dependent G2 checkpoints, may be targeted by mitotic checkpoint inhibitors such as Mps1 inhibitors that elicit an accumulation of mitotic defects resulting in more unstable and less fit karyotypes (Cohen-Sharir et al., 2021). If not driving catastrophic outcomes, would this combination be a driver of neo-antigen production prescribing immuno-oncology approaches?

## Perspectives on Topo II checkpoints

There are many outstanding questions associated with the operation/inaction of these Topo IIA–dependent checkpoints and delays that have implications not only for our understanding of the mechanistic details but also for the deployment of interventions that can exploit the idiosyncratic dependencies of transformed cells. Some of these issues we have highlighted above, among others, is the question of timing—in G2, the engagement of a long-term arrest and commitment to a senescence program is not hard-wired, with some transformed cells delaying transit but then progressing through G2- to M-phase, albeit in a passage-dependent manner (Kaufmann et al., 2002). What dictates this pattern of behavior, how is the transient delay implemented, and why does this erode on passage—what selective advantage does this confer? There is also the question of the origins of these pathways. Given the existence of a series of conditional control processes operating in M-phase and then anaphase, what can we conclude about the evolution of these processes? Assuming these did not arise simply to enable transformed cells to circumvent the Topo IIA–dependent G2 checkpoint, it seems likely that there are physiological contexts in which normal cells are programmed to avoid this p53-dependent G2 checkpoint while retaining some ability to reduce the undesired consequences through the ensuing mitotic and abscission checkpoints. It is interesting that in mouse stem and progenitor cells, there is a deficiency in this checkpoint, and this is recovered when embryonic stem cells are differentiated in response to retinoic acid (Damelin et al., 2005). It is hard to rationalize this loss of checkpoint control in cell types crucial to tissue homeostasis where there is a requirement for complete integrity in cell division. It would be of interest to determine whether replication in these cells relied exclusively on Topo I acting ahead of the replication fork to obviate the need for Topo IIA action after replication. By contrast, there are circumstances where a lowered tolerance in the requirement for precision might be more easily rationalized; for example, in a pathogen response where the demand for the very rapid adaptive immune cell expansion of pathogen-recognizing clones overrides the more time-demanding process of high-fidelity cell division, sacrificing some degree of genomic integrity in a life-and-death struggle (Bonilla and Oettgen, 2010). A similar rationalization would apply in the context of hyperplasia associated with response to (life-threatening) injury.

In bypassing the Topo IIA–dependent G2 checkpoint, cells engage control processes that are not usually required for cell cycle regulation. This is well characterized for the engagement of PKCε in the Topo IIA–dependent mitotic delay, where the reliance is not a function of somatic mutation as pharmacological bypass in otherwise G2-arrest competent cells still engages PKCε (Brownlow et al., 2014). It seems likely that this reflects the acute, stress-specific implementation of distinct cell cycle regulatory programs. Related to this, there is no evidence of PKCε engagement in the abscission checkpoint, except in cells with Topo IIA involvement; similarly, there is no PKCε engagement in replication stress-driven behaviors in mitosis, despite there being non-disjunction challenges in the context of collapsed replication forks and incomplete DNA replication (Lin and Pasero, 2021). So what triggers and maintains the selective downstream program, and are the ultimate targets of these programs common across cell cycle–associated stresses? Some indication of downstream overlap comes from the finding that the p53–p21 axis is a target of the G2 Topo IIA checkpoint pathway in common with one of the redundant DDR pathways triggered in G2 (Lockwood et al., 2022). Equally, it is unknown how different Topo IIA–associated stress-induced regulatory programs delay SAC silencing, activate Topo IIA, modify mitotic translation, and ultimately regulate exit from the abscission checkpoint. Resolution of these issues will offer precision approaches to interventions.
